# Do Past Psychiatric Conditions or Pre-Existing Vulnerabilities Influence the Neuropsychiatric Presentation in People with Neurological Conditions?

**DOI:** 10.1192/bjo.2026.11058

**Published:** 2026-06-30

**Authors:** Soracha Healy, Niruj Agrawal, Bruce Tamilson, Alexander Benson

**Affiliations:** South West London and St George’s NHS Mental Health Trust, London, United Kingdom

## Abstract

**Aims:**

To establish whether past psychiatric conditions or pre-existing vulnerabilities influence the neuropsychiatric presentation in people with neurological conditions, and to identify common reasons for referral and diagnoses to help direct service delivery and enhance patient care.

**Methods:**

A retrospective study of consecutive inpatient Neuropsychiatry referrals from the Neurosciences wards at the Atkinson Morley Regional Neurosciences Centre at St George’s Hospital, London, was conducted. All referrals from 1 January 2022 to 1 January 2024 were reviewed. The exclusion criteria were duplicate referrals and patients who were not seen by Neuropsychiatry following referral. Reasons for this included being transferred or discharged before being seen or clinically inappropriate referrals.

A data collection proforma was designed and trialled on a small sample of records to identify any issues before being implemented for wider use. Data was extracted from the clinical notes systems, RiO and iClip, and collected using RedCAP, an online secure data collection system, with pseudo-anonymisation.

**Results:**

362 records were reviewed and 307 included. The main reasons for exclusion were duplicate records (19), other (18), transferred before being seen (10), inappropriate referral (7), and died before being seen (1). The age range of patients was from 18–100 years with a mean age of 53 years with 58.0% biologically male and 42.0% biologically female.

Referrals were predominantly from the regional neurology ward (41.4%), followed by neurosurgery (23.8%) and the hyperacute and acute stroke units (21.4%). Reasons for referral were varied with the most common being mood (45.0%), agitation/aggression (30.0%) and suicidality (24.1%).

Following assessment, low mood was the most reported symptom occurring in 65.1% of referrals followed by sleep (58.6%), anxiety (45.6%) and agitation (31.6%). Differences were seen in the most common symptom depending on the primary neurological diagnosis. Traumatic Brain Injury (TBI) had the strongest association with agitation, Cerebrovascular Accident (CVA) with depression and Functional Neurological Disorder (FND) with motor disturbance and anxiety.

Those with a psychiatric history (56.4%) were more likely to present with low mood (70.0%) whereas those without were more likely to present with sleep problems (64.1%). This pattern was not seen in those with a history of trauma (26.7%). A high mortality rate was observed with 14.7% of patients deceased within 2 years of referral.

**Conclusion:**

This research provides valuable insights into the complex, bidirectional associations between neurological and psychiatric conditions. These insights will help to identify areas for improving service delivery and therefore enhancepatient care.

